# Wireless, Multifunctional System-Integrated Programmable Soft Robot

**DOI:** 10.1007/s40820-024-01601-3

**Published:** 2025-02-17

**Authors:** Sungkeun Han, Jeong-Woong Shin, Joong Hoon Lee, Bowen Li, Gwan-Jin Ko, Tae-Min Jang, Ankan Dutta, Won Bae Han, Seung Min Yang, Dong-Je Kim, Heeseok Kang, Jun Hyeon Lim, Chan-Hwi Eom, So Jeong Choi, Huanyu Cheng, Suk-Won Hwang

**Affiliations:** 1 KU-KIST Graduate School of Converging Science and Technology, Korea University, 145 Anam-Ro, Seongbuk-Gu, Seoul, 02841 Republic of Korea; 2 Semiconductor R&D Center, Samsung Electronics Co., Ltd., Hwaseong-Si, Gyeonggi-Do 18448 Republic of Korea; 3 SK Hynix, Icheon, 17336 Republic of Korea; 4 Department of Engineering Science and Mechanics, The Pennsylvania State University, University Park, PA 16802 USA; 5 Center for Neural Engineering, The Pennsylvania State University, State College, University Park, PA 16802 USA; 6 Hanwha Systems Co., Ltd., 188, Pangyoyeok-Ro, Bundang-GuGyeonggi-Do, Seongnam-Si, 13524 Republic of Korea; 7 Department of Materials Science and Engineering, The Pennsylvania State University, State College, University Park, PA 16802 USA; 8 Materials Research Institute, The Pennsylvania State University, State College, University Park, PA 16802 USA; 9 Center for Biomaterials, Biomedical Research Institute, Korea Institute of Science and Technology (KIST), Seoul, 02792 Republic of Korea; 10 Department of Integrative Energy Engineering, Korea University, Seoul, 02841 Republic of Korea; 11 Department of Mechanical Engineering, The Pennsylvania State University, University Park, PA 16802 USA

**Keywords:** Untethered multimodal locomotion, Soft robotics, Soft electronics, Wireless, Reprogrammable magnetic soft robot

## Abstract

Soft robots have partially or entirely provided versatile opportunities for issues or roles that cannot be addressed by conventional machine robots, although most studies are limited to designs, controls, or physical/mechanical motions. Here, we present a transformable, reconfigurable robotic platform created by the integration of magnetically responsive soft composite matrices with deformable multifunctional electronics. Magnetic compounds engineered to undergo phase transition at a low temperature can readily achieve reversible magnetization and conduct various changes of motions and shapes. Thin and flexible electronic system designed with mechanical dynamics does not interfere with movements of the soft electronic robot, and the performances of wireless circuit, sensors, and devices are independent of a variety of activities, all of which are verified by theoretical studies. Demonstration of navigations and electronic operations in an artificial track highlights the potential of the integrated soft robot for on-demand, environments-responsive movements/metamorphoses, and optoelectrical detection and stimulation. Further improvements to a miniaturized, sophisticated system with material options enable in situ monitoring and treatment in envisioned areas such as biomedical implants.

**Supplementary Information:**

The online version contains supplementary material available at 10.1007/s40820-024-01601-3.

## Highlights

- A soft, untethered electronic robot that integrates magnetically responsive engineered composites, enabling reversible programming and a diverse range of motions and shapes.
- Seamless integration of a meticulously designed soft/flexible electronic system with the magnetic soft robot guarantees the stable and accurate execution of multi-modal electrical functions while preserving the integrity of its mechanical movement.
- The comprehensive demonstration illustrates the feasibility of the untethered, integrated magnetic soft robot, highlighting its stable operation, adaptability, and ability to perform complex tasks under diverse conditions.

**Supplementary Information:**

The online version contains supplementary material available at 10.1007/s40820-024-01601-3.

## Graphical abstract

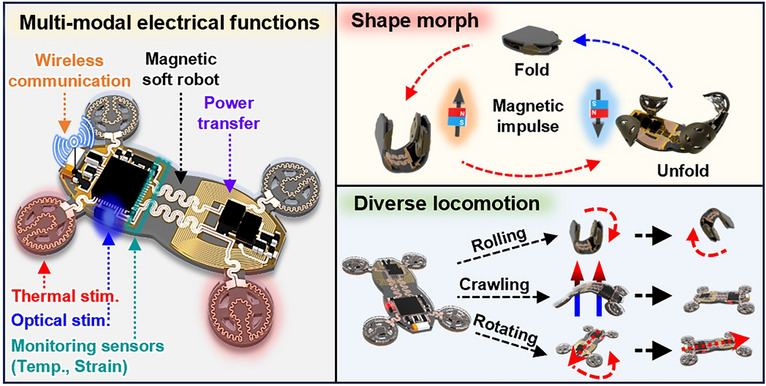

## Introduction

Soft, flexible platform technology created research in diverse, envisioned fields based on findings of innovative materials and structures that enable to form defect-free, seamless interfaces to the human body, which offered precise detection of physiological parameters or provided useful functions through delivery of drugs and stimuli. Exampled studies are broadly categorized into non-invasive and invasive types: The former includes electronic skin (E-skin) [1–4], personal healthcare [5–7], augmented reality/virtual reality [8, 9], and human–machine interface [10, 11], and the latter involves drug delivery, stimulation, optogenetics, and medical implants (bioresorbable) [12–15].

Such technology transition with mechanical elasticity has also been extended to robotics. Unlike conventional robots that rely on metallic or plastic joints and motor-driven linkages, mechanically soft robots enabled to interact with living organisms or delicate objects and provide deformable, biologically inspired movements including navigation in complex and unfamiliar environments [16, 17]. While there are many ways to drive the unusual robots, particularly non-tethered, un-restrained soft robots that can freely move in response to various external stimuli—temperature, light, chemical reaction, and electric and magnetic field—have been proposed [18, 19]. Among these stimuli, magnetic field-induced actuation offers the favorable ability to pass through enclosed spaces such as the internal environment of the human body without any harmful effects or reactions [20]. The ease and precise control of magnetic fields in terms of phase, frequency, and magnitude, facilitated by the development of advanced manipulation systems [21–23], led to significant advances in the design, fabrication of magnetic soft robots, enabling diverse motions such as crawling, rolling, jumping, spinning, and swimming [24–35]. Despite such advances, the operation of magnetic soft robots is still limited to simple movement and/or actuation, restricting the ability to perform a wide range of tasks. Beyond the passive operation to regulate movements, there might be opportunities to achieve functions including perception, detection, or active stimulation through integration of functional modules for sensing surroundings [36–38] and thermal stimulation [39]. While these approaches are promising, they often require module replacement and/or face actuation limitations due to the mechanical mismatch between rigid/large modules and soft robots. In this respect, a magnetic soft robot with integrated flexible electronics provides the ability to enhance and expand the functionality via satisfying unmet demands including environmental detection, electrical/thermal/chemical actuation, and remote control and interaction through wireless communication.

In the following, we introduce a soft electronic robot that seamlessly combines a magnetically responsive robotic platform with advanced electronic modules. The engineered soft composites are reversibly programmable at a low temperature and allow for modes of actuations, transformations, and locomotion of the integrated robotic system. The electronic system layouts enable the accurate execution of various electrical functions without interference with mechanical motions of the magnetic soft robot. Demonstration in an artificial racetrack validates the possibility of stable operation and adaptability, and elaborated tasks under diverse conditions, potential applicable for environmental monitors or medical implants using biosafety materials with miniaturization of the whole system.

## Experimental Section

### Synthesis of the Wax-Coated Magnetic Particles (WcMPs)

NdFeB (average ~ 1 µm, Nanochemazone Inc., Canada) microparticles (MPs) were treated by trichloro(octadecyl)silane (OTS, Sigma-Aldrich, USA) on the 80 °C hotplate for 2 h to obtain hydrophobic magnetic MPs. The OTS-treated NdFeB MPs were mixed with molten soybean wax (GW 464, AAK, USA) heated at 60 °C. Following this step, 1 g of NdFeB@wax (WcN; wax-coated NdFeB) droplet and 20 mL of polyvinyl alcohol (PVA, Sigma-Aldrich, USA) aqueous solution as a separator on the 60 °C hotplate were stirred at 1500 rpm for 5 h to ensure homogeneous mixture and then cooled to room temperature (RT) rapidly in the ice bath for 1 min, promoting the encapsulation of the magnetic particle within the wax capsule. The produced WcN MPs were rinsed with deionized (DI) water several times to remove PVA on the surface. Then, WcN MPs were dried at room temperature for a day to obtain wax-coated magnetic particles (WcMPs, average ~ 10 µm).

### Preparation of Programmable Magnetic Soft Composites

Programmable magnetic composite was prepared by mixing the synthesized WcMPs with a silicone elastomer and curing agent (Ecoflex 00–30, Smooth-On, USA) in a 1:1:1 mass ratio, poured onto the Teflon plate, and cured at RT for 3 h. Then, the cured magnetic soft composites (~ 500 µm thick) were variously patterned using the CO_2_ laser (Epilog Laser, USA). The patterned magnetic soft composites were programmed by heating above 45 °C to melt wax capsules, applying an external magnetic field (200 mT) to rotate and align the direction of embedded magnetic particles, and then cooling down to RT to immobilize the particles.

### Characterization of the Magnetic Composites

Surface morphologies of the WcMPs were observed with an optical microscope (BX53M, OLYMPUS, Japan). Mechanical tensile test was performed with a universal testing machine (Instron 5900 series, USA) by applying uniaxial strain to specimens (length: 10 mm, width: 5 mm) at a consistent loading rate of 6 mm min^−1^ with varying thickness. Magnetic flux density of the magnetized matrix was measured by a gauss meter (TM-197, TENMARS, Taiwan), while magnetic hysteresis loop of the encoded matrix was evaluated using a SQUID-vibrating sample magnetometer (SQUID-VSM, Quantum Design Inc., USA).

### Fabrication of Integrated Magnetic Soft Robot

Electronic device fabrication began with a coating of the bottom sacrificial/encapsulation layer of poly (methyl methacrylate) (PMMA, Microchem, USA)/polyimide (PI, Sigma-Aldrich, USA) on the copper (Cu) foil (~ 3 µm thick, Nilaco, Japan). The coated Cu foil was transferred onto a polydimethylsiloxane (PDMS, Dow corning, USA) slab. The Cu was photolithographically patterned to form a radio frequency (RF) coil (trapezoid shaped, upper/lower base: 5 mm/10 mm, height: 10 mm, and 10 turns). A layer of PI (~ 5 µm thick) was coated for interlayer dielectrics (ILDs) on top of an RF coil, followed by the opening of the ILDs for interlayer connection that was processed using oxygen reactive ion etching (RIE, JVAC, Korea) with the following conditions: pressure at 200 mTorr, oxygen flow at 25 sccm, and 150 W of RF source. A layer of Cu (~ 5 µm thick) as interconnects and electrodes was deposited by an electron-beam evaporator (E-beam, Korea Vacuum Tech, Korea). After that, the top encapsulation layer of PI (~ 2 µm thick) was coated, dry etching and immersion in acetone-enabled transfer printing of the entire device and opening for contact pads. A thin layer of SiO_2_ (~ 5 nm thick) as an adhesion layer was deposited on the back side of the device as an adhesion layer using a thermal evaporator (DK110319-3, DKVAC, Korea). After SiO_2_ was coated, the entire device was transferred onto the programmable magnetic soft matrix (~ 500 µm thick). The electrical components (microcontroller, diodes, capacitor, µ-LEDs, BLE SoC, and Bluetooth antenna) were assembled onto the contact area by a silver epoxy with curing at RT for 2 h. Finally, the top of the entire system was encapsulated with a layer of Ecoflex 00–30 (~ 100 µm thick) to complete the fabrication of the integrated magnetic soft robot.

### Manipulation of Magnetic Soft Robot

For all actuation performances presented in this paper, a commercial permanent NdFeB magnet (N52, maximum 200 mT, 50 mm × 50 mm × 25 mm) and customized cylinder electromagnet (maximum 400 mT, 100 mm diameter, 60 mm thick) were used to apply the external magnetic field required for locomotion of magnetic soft robot. The strength of the external magnetic field (0 ~ 200 mT) applied to the robot can be adjusted by varying the distance (0 ~ 20 cm) between the magnet source and the soft robot. Various frequencies (0 ~ 2.5 Hz) of magnetic fields can be generated by modulating the current flowing through the electromagnet using relay module. Performance of each locomotion of the soft robot was controlled by the combination of various 3-dimensional manipulations (where x-direction indicates forward, crawling: sinusoidal waveform in x/z-axes, rolling: rotation along x/y-axes, rotation: revolution around the robot along z-axis) and frequency modulation of the external magnetic source.

### Evaluation of the Stability of the Electrical Components under External Magnetic Field

Performance stability of electrical components, including inductor, temperature, strain, μ-heater, microcontroller (μC), diodes, transistors, capacitors, μ-LED, and Bluetooth low-energy system-on-chip (BLE SoC), due to exposure to magnetic fields was evaluated under various ranges of external magnetic fields (0 ~ 200 mT). The real-time resistance changes in the number of device actuation trials (N = 1000) by cyclic test were measured using a source meter (Keithley 2636b, USA). The efficiency of the wireless power transfer of an RF coil was examined by a vector network analyzer (MS 2024 A, Anritsu, Japan). Current–voltage (I-V) characteristics of the temperature/strain sensor were measured by a source meter and recorded by LabVIEW software (National Instruments, USA), while the diode/transistor were evaluated by a mechanical probe station (HP 4145B, Hewlett-Packard, USA). Heating capability of µ-heater was evaluated by applying voltage to the heater using a power supply and observing temperature changes with an infrared (IR) thermal imaging camera (Fluke Ti480 PRO, Fluke, USA). Investigations of changes in optical intensity and capacitance were recorded by a digital optical power meter (PM 100D, Thorlabs, USA) and LCR meter (IM 3533-10, HIOKI, Japan).

## Results and Discussion

### A Remotely Operated, Ferromagnetic, Soft Robotic System

**Fig. 1:**
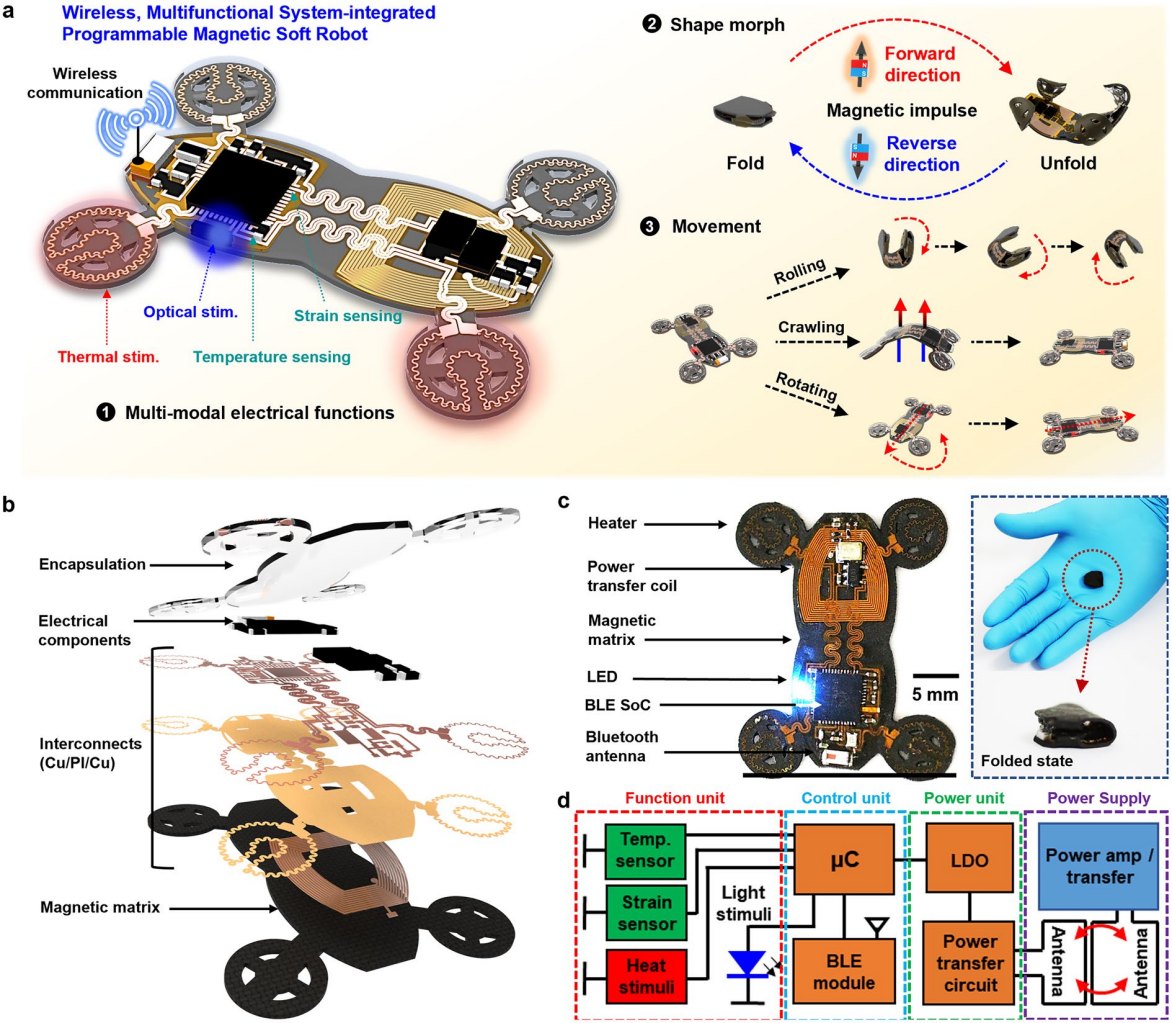
Materials, design, components of the integrated magnetic soft robot system. **a** Schematic illustration of the integrated magnetic soft robot capable of wireless multi-modal electronics, magnetically controlled reversible shape transformation/reconfiguration, and untethered versatile locomotion. **b** Exploded-view drawing of a complete system, comprising a soft encapsulation layer (Ecoflex 0030, ~ 100 µm thick), electronic components, flexible interconnects with copper/polyimide/copper (Cu/PI/Cu, 3/5/3 µm thick), and a ferromagnetic elastomeric matrix (~ 500 µm thick) incorporated with wax-coated neodymium-iron-boron (NdFeB) microparticles (average size, ~ 10 µm). **c** Photograph of a representative example of the integrated magnetic soft robot, equipped with a heater, radiofrequency (RF) antennas, microscale light-emitting diodes (µ-LEDs), sensors, Bluetooth low-energy system-on-chip (BLE SoC), and Bluetooth antenna. **d** Block diagram of the overall electronics, with units of a wireless power supply, power management, control, and functions

Figure 1a presents a schematic illustration of a soft, untethered robot with the ability to perform versatile modes of locomotion and electronic functions. The key aspects of the platform involve: (1) multi-modal functions with wireless power supply—monitoring physical parameters such as temperature and strain, transmitting data to smart devices, and providing thermal/optical stimulation as required; (2) shape morphing via external magnetic field—programmed magnetic domains of a ferromagnetic soft matrix; and (3) untethered, versatile locomotion—traveling across different terrains including a bumpy ground and water (Fig. S1), via crawling, rolling, and rotating. Figure 1b illustrates an exploded-view drawing of the integrated magnetic soft robot that consists of: (bottom) a motor vehicle-inspired ferromagnetic soft matrix built with a mixture of soft silicone rubber (Ecoflex 0030, ~ 500 μm thick) and wax-enclosed neodymium-iron-boron microparticles (NdFeB, ~ 1 μm) to actuate and change shape into diverse modes; (bottom-middle) layers of flexible copper (Cu)-based radio frequency (RF) power transfer coil (~ 3 μm thick; resonant frequency, 6.78 MHz), dielectric polyimide (PI, ~ 5 μm thick), and Cu interconnects (~ 3 μm thick); (top-middle) electronics with commercial off-the-shelf (COTs) components such as a microcontroller (μC), Bluetooth low-energy system-on-chip (BLE SoC), chip antenna, linear dropout regulator (LDO), diodes, capacitors, resistors, and microscale light-emitting diodes (μ-LEDs); and (top) soft encapsulant (Ecoflex 0030, ~ 100 μm thick) to protect whole electronic components against wet and dry conditions and various deformations. Figure 1c shows a representative example of shape-deformable, integrated magnetic soft robot and an image at a folded state. The functional block circuit diagram in Fig. 1d indicates the overall system procedures with wireless operation to regulate sensors/actuators, convert analog to digital data, and communicate remotely via Bluetooth to external devices. Additional information on materials and fabrication appears in the Experimental section.

### Fabrication, Characterization, and Manipulation of Magnetic Soft Composites

**Fig. 2:**
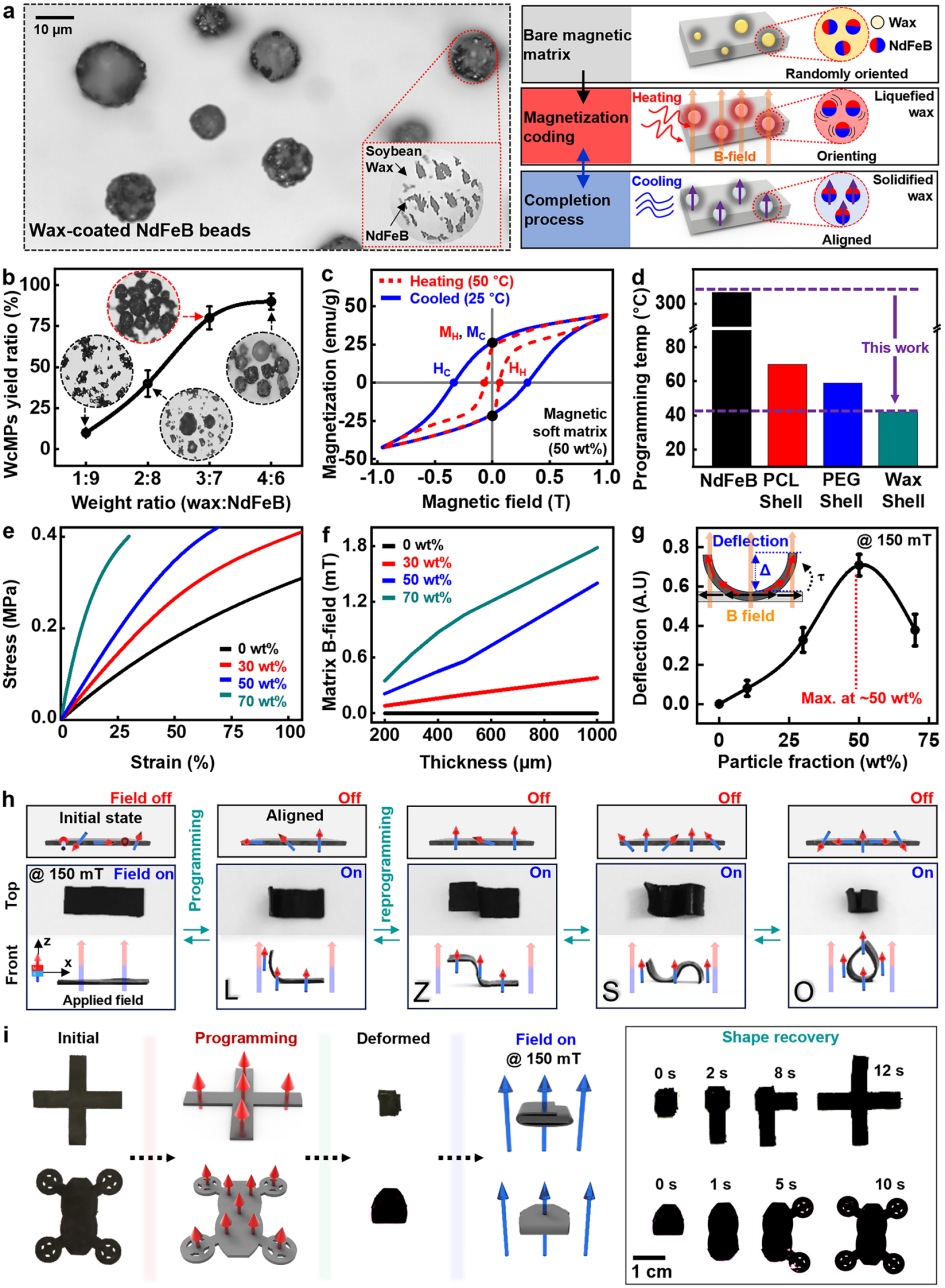
Characterization of magnetic soft composites and actuation performance. **a** Optical image of soy wax-coated NdFeB microparticles (WcMPs) with an enlarged view of the bead (left), and reprogrammable magnetization process (right): increase in temperature (~ 45 °C) (top); manipulation of magnetic moments of NdFeB microparticles (middle); and decrease in temperature to complete the process (bottom). **b** Evaluation of WcMPs yield, depending on weight ratio of soybean wax and NdFeB particles, and images of obtained WcMPs in the inset. **c** Measurements of magnetic properties of the soft polymeric composites. **d** Comparison of magnetization temperatures among the reported NdFeB and polymer shells including polycaprolactone (PCL) [28], polyethylene glycol (PEG) [40], and wax shell. **e** Mechanical behaviors of the ferromagnetic soft elastomers with different weight concentrations of WcMPs under uniaxial strains. **f** Measured changes of B-field strength of ferromagnetic soft elastomers with weight concentrations of WcMPs at varying thicknesses. **g** Deflection at both free ends of the magnetic soft elastomers (~ 500 μm thick) as actuation performance, depending on particle volume fractions. **h** Reversible, diverse transformations of the magnetized matrices via external magnetic field (B-field; 150 mT). The arrows indicate distribution of magnetized orientation of the matrix (red, north pole; blue, south pole). **i** Capability of regulating various distinct shapes of the magnetized soft matrices in complex deformations via static B-field (150 mT)

For the actuation of magnetic soft robots, the internal magnetic domains must be aligned, and thus various reports introduced programming strategies such as applying external magnetic fields while curing magnetic composites [26, 34, 40], heating materials above the Curie temperature using lasers or thermal sources for temporary demagnetization followed by re-magnetization [28, 29], and programming materials with a magnetic field during additive manufacturing [25, 30]. While these approaches are still useful and effective, they require a significant amount of thermal energy for demagnetization, potentially causing physical deformation to the soft robots. Therefore, control of the preferred orientation of magnetic particles at low temperatures is crucial for various operation modes. Previous studies utilized phase transition materials such as polycaprolactone (PCL) [28] and polyethylene glycol (PEG) [40], even alloys [41] to lower the programming temperature, while a wide range of material options may be required for application in diverse research fields. Figure 2a displays images of programmable wax-coated magnetic particles (WcMPs, average size ~ 10 μm) (left) and procedures to control or manipulate a preferred magnetic orientation (right). The particles consist of soybean wax as a phase change material and NdFeB as a hard magnetic substance, and the magnetic orientation can be tuned under external fields near the transition temperature of the wax (~ 45 °C). We note that the transition temperature of wax can be adjusted by blending with other waxes, supporting its potential applicability in various temperature conditions [42–44]. Details of the process appear in Experimental section and Method S1 and Fig. S2. Figure 2b shows yield of WcMPs varying with the mixing ratio of soybean wax and NdFeB particles. As the portion of the wax increased, the number of embedded particles also increased, but above the 4:6 ratio, the wax fraction per WcMPs is high, resulting in it being difficult to respond to external magnetic field. Figure 2c exhibits magnetic behaviors of the soft composites during the programming process. Both procedures (heating, cooling) indicated similar magnetization values due to the inherent property of the embedded magnetic particles (MC: 28.9 emu g^−1^, MH: 26.5 emu g^−1^), while temperature-dependent coercivity offered easy control and retention in the presence of external fields. Figure 2d provides comparison of programming temperatures of several conditions including NdFeB particles, and the particles coated with PCL [28], PEG [40], and soybean wax, which demonstrated that use of the wax shell can minimize thermal energy required for magnetic reorientation and prevent thermal damage to the polymer matrix. Figure 2e shows stress–strain curves of magnetic soft composites as per concentration of WcMPs. As the content of the WcMPs increased, the mechanical rigidity proportionally increased as expected; however, the robot’s degree of freedom in deformation may decrease due to a reduction in mechanical flexibility. Figure 2f displays thickness-dependent magnetic fields with different WcMPs fractions. Thicker robots exhibited stronger magnetic responses than thinner robots although precise control over movements may be the opposite. Figure 2g exhibits the ability to actuate square beam-shaped magnetized composites (~ 500 μm thick, 20 mm long) at different fractions of WcMPs under uniform external magnetic field (150 mT) to the vertical direction. As WcMPs concentration increased, responses to magnetic stimuli increased to a certain critical point (50 wt%. ~ 0.207 volume fraction) and then decreased due to elevated mechanical stiffness of the magnetic composites, which agrees well with the theoretical model. Details of the theoretical calculation appear in Method S2 [25, 45–47]. Figure 2h illustrates versatile profiles of magnetic soft composites via reprogrammable magnetization process. Arrows in the images (top) show local magnetic moments of the particles in the polymer matrix, with the red and blue color pointing to the north and south poles, respectively. A composite with randomly distributed moments exhibited no changes of shape under an applied external field, while the reprogrammable process was able to control the magnetic moment in diverse modes for multiple shape transformations including letters such as L, Z, S, and O. The cyclic results over 100 times in Fig. S3 indicate that the magnetic flux density was well-maintained without significant changes, resulting in excellent repeatability and stability of programming process of the magnetic soft robots. Figures 2i and S4, Movie S1 show examples of the ability to manipulate a shape recovery process via external magnetic field. Magnetically encoded composites of cross and automobile-like models were completely folded and restored to the original shapes within ~ 10 s after applying the external field (150 mT), which suggests the possibility of shape restoration in complex three-dimensional (3D) deformations.

### Experimental and Theoretical Evaluations of the Integrated Magnetic Soft Robots on Versatile Modes of Locomotion

**Fig. 3:**
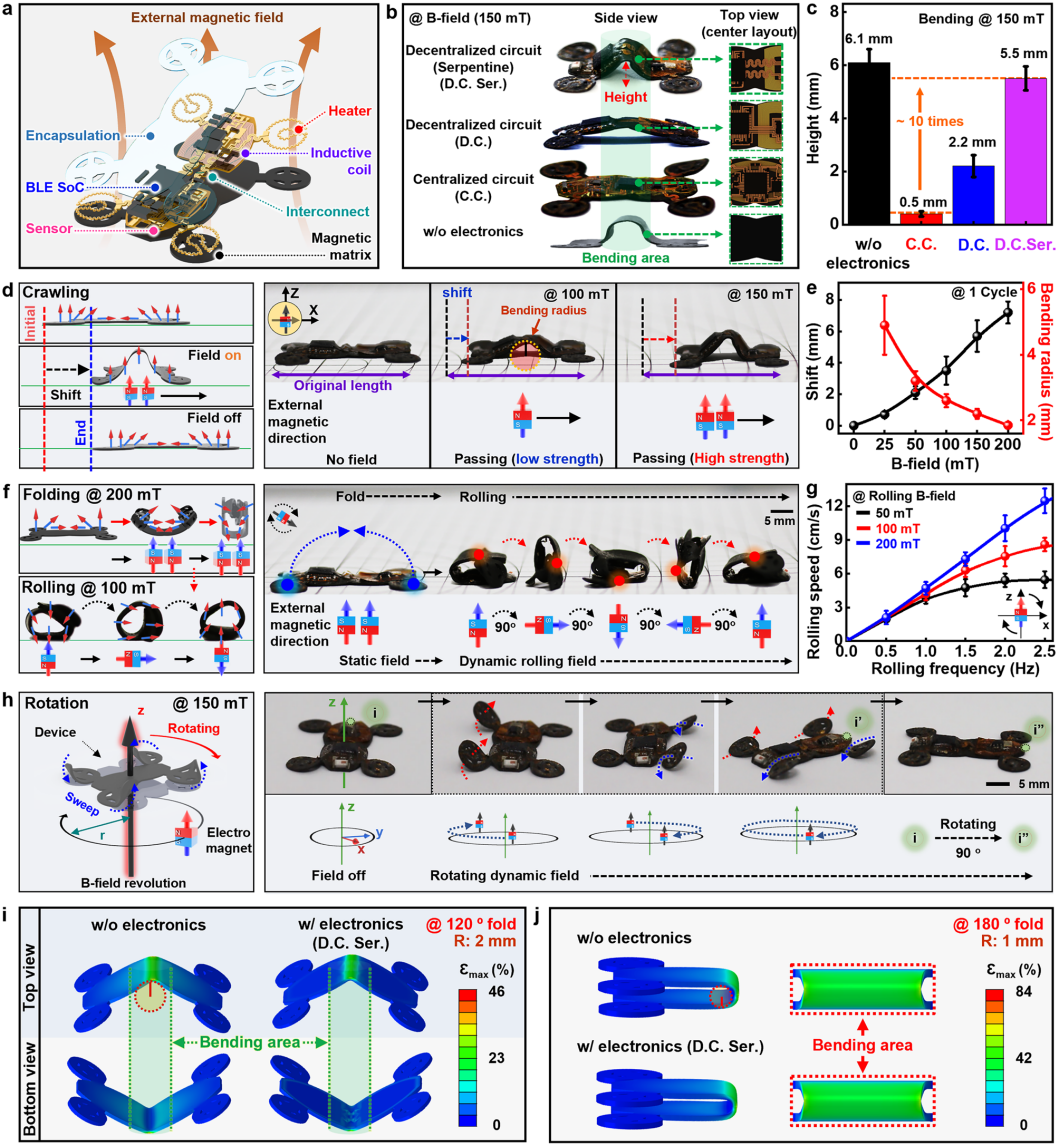
Integration of electronics on magnetic soft robot and studies of their locomotion. **a** Schematic illustration of an electronics-integrated, deformable magnetic robot capable of diverse electrical functions and versatile locomotion. **b, c** Demonstration of relative height difference at bending area of magnetic robot showing crawling locomotion according to electronic circuit layouts (**b**) (w/o electronics: magnetic matrix, C.C.: centralized circuit, D.C.: decentralized circuit, and D.C.Ser.: decentralized circuit with serpentine interconnect) when applied B-field of 150 mT and evaluation of height difference (**c**). **d** Fundamental procedures of crawling motion of the integrated magnetic soft robots in response to external B-field source. **e** Measured changes in travel distance and bending radius actuated by magnetic field strengths. **f** Control of folding and rolling motions of the integrated magnetic soft robots via different modes of magnetic fields. **g** Measurements of rolling speed of the robots as a function of rolling frequency of applied B-field. **h** Rotational motion of the soft robots to change or navigate direction via individual wheels modulated by different modes of magnetic fields. **i** Top and bottom view of strain distribution for robot system folding at 120-degree with the radius of curvature at 2 mm. **j** Strain distribution for robot system folding at 180-degree with the radius of curvature at 1 mm

Integration of a thin and flexible electronic system with a passive soft robot provides a variety of functions that can perceive environmental cues and/or apply stimulation as a feedback control (Fig. 3a). Here, the electronics was designed to minimize the influence on various modes of locomotion by distributing circuit and commercial chips in a balanced manner and configuring the interconnects in a serpentine pattern, since the integration might cause mechanical interruption or hindrance to actuations or motions of the soft robots. We tested actuation performance of integrated soft robots in Figs. 3b and S5, and Movie S2 with different system layouts (w/o electronics: magnetic matrix, C.C.: centralized circuit, D.C.: decentralized circuit, and D.C.Ser.: decentralized circuit with serpentine interconnect) under external field (150 mT), and comparison of measured heights in bending region is described in Fig. 3c. The results indicate that D.C.Ser. can be deformed over 10 times more than C.C. layout, exhibiting similar behavior to that of the pristine magnetic soft robot. Furthermore, the deformation of the integrated magnetic soft robot exhibited a consistent level of actuation performance even after four weeks, as can be found in Fig. S6, indicating the stable long-term operation. Crawling behaviors of the integrated soft robot in terms of bending radius or stride length can be controlled by modulating the magnetic field strength (Fig. 3d), with quantitative measurements conducted over a broad range of magnetic fields (0 ~ 200 mT) at a frequency of 1 Hz (Fig. 3e). As a result, changes in the intensity (0 ~ 200 mT) and frequency (0 ~ 2.5 Hz) of the magnetic field led to the control in crawling speed (Fig. S7) and sequential rolling (Fig. 3f). After upward bending and complete folding in the magnetic field (200 mT), the integrated robot underwent forward rolling along the x-direction in response to clockwise rotation of the external field (100 mT) along the z-axis. Figure 3g summarizes the resulting correlation between rolling performance and rotation frequencies of the magnetic fields. The rolling speed was proportional to the rotation frequency of the magnetic field and then gradually saturated, particularly in low strength fields (50 mT) due to insufficient magnetic responsiveness [34, 40, 48]. The rolling motion can also be reversed or steered depending on the direction of the magnetic field (Fig. S8). With four wheels placed on each corner of the integrated soft robot to yield an automobile-like object, the system can further manipulate the navigation path (Fig. 3h). Revolution of external magnetic fields can switch or turn the trajectory of the system in paddling-like motions, with the navigational mode affected by the radius and speed of the external magnetic field (Fig. S9). Furthermore, we validated the manipulation reliability of the integrated magnetic soft robot by conducting repeated trajectory tracking tests for each locomotion including crawling, rolling, and rotation (Fig. S10). Considering the design layouts in Figs. 3b, c and S5, the soft robots with and without electronic circuits over a radius of curvature of 1 mm (bending angle, 180-degree) and 2 mm (bending angle, 120-degree) were simulated with commercial software (Dassault Abaqus 2022). The maximum principal bending strain in the soft robot without electronic circuits was ~ 20% higher than that with electronics (Fig. 3i). Compared with the centralized circuit, the decentralized circuit on the soft robot exhibits ~ 150% lower bending strain for the same bending radius of curvature of 2 mm (bending angle, 120-degree) (Fig. S5b and c), demonstrating the advantage of the distributed circuit design. Also, compared to the bending of 120°, the bending of 180° here shows an increase of ~ 65% in the maximum principal strain despite a similar strain distribution map (Fig. 3j).

### Experiments and Theoretical Considerations on the Mechanical Stability of the Integrated Soft Robots and Electrical Characteristics of Devices under Magnetic Forces

**Fig. 4:**
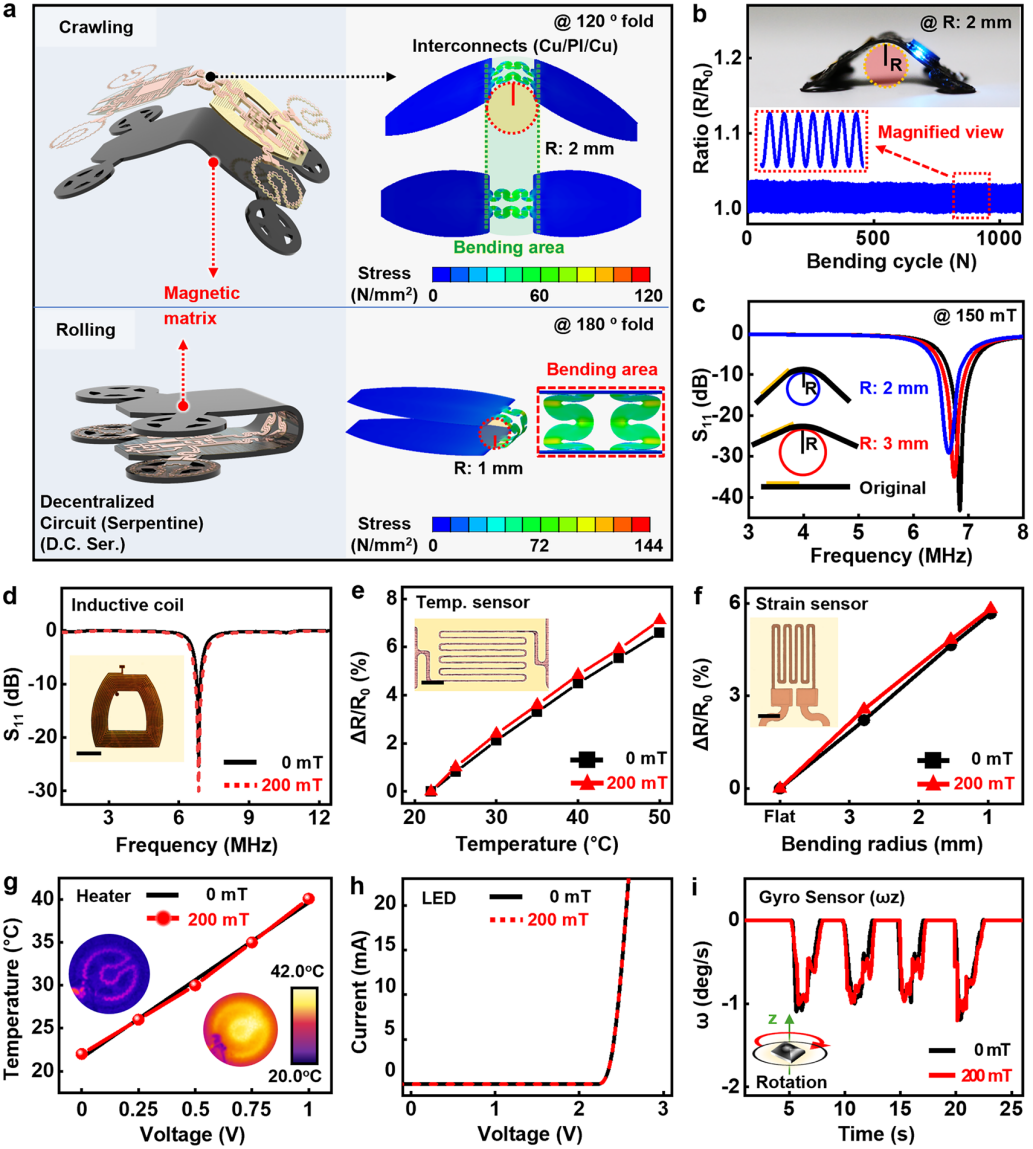
Electrical and mechanical stability of integrated magnetic soft robot under applied B-field. **a** Electronic part stress distribution for robot system at crawling (folding: 120-degree, radius: 2 mm) and rolling (folding: 180-degree, radius: 1 mm) status. **b** Resistance changes of electrical interconnects in the system during repeated bending tests at a radius of ~ 2 mm over 1000 times (B-field, 150 mT), with an image of wireless operation in a deformed condition. **c** Scattering spectra (S11) of wireless power transfer coils at different bending radii (flat to 3 mm). **d** Effect of peripheral magnetic fields on S11 of Cu inductive coil (resonance frequency: ~ 6.8 MHz). **e, f** Measured fractional changes of resistance in temperature (**e**) (range: 20–50 °C) and strain sensors (**f**) (bending radii: flat to 3 mm) with and without external B-field. **g** Heating performance of microheaters under the presence of applied B-field, and infrared (IR) images before and after the operation in the inset. **h** Comparison of I-V characteristics of μ-LEDs under B-field. **i** Evaluation of the effect of a magnetic field on the performance of gyro sensors

It is also vital to assess the influences of the magnetic fields or field-induced mechanical motions on the mechanical and electrical properties of individual electronic devices. The field-induced stress distribution in the electronic parts on the integrated robot indicates stress concentration on the inner side of the center in serpentine structures for both 120° (with radius of curvature of 2 mm) bending and 180° (with radius of curvature of 1 mm) folding (Fig. 4a). The measured electrical resistance in the interconnects with a lighted blue LED shows negligible changes upon repeated bending with a radius of 2 mm over 1000 times (Fig. 4b), demonstrating excellent durability. The measured changes in S_11_ spectra of power transfer coils slightly increased with distance from the primary coil (Tx, transmitting coil) located on the ground upon bending (Fig. 4c), but such an issue could be minimized through optimized design of the Tx coil as approaches in previous reports [49, 50]. Wireless power receiver coils under the magnetic interferences exhibit slightly enhanced efficiency in scattering parameters (S_11_) (Fig. 4d), open-circuit voltages, and generated voltages at a given working distance (Fig. S11). The enhancement could be attributed to the increased mutual inductance between transmission and receiver coils from ferromagnetic materials in the magnetic field [51, 52]. Besides minimized changes in the measured temperatures, strains (Fig. 4e and f), and capacitances (Fig. S12), the efficient heating behavior from resistive microheaters under the presence of applied magnetic fields was also confirmed by the embedded infrared (IR) images (Fig. 4g). Semiconducting materials (e.g., Si, Ge, GaAs, and GaP) to serve in active electronic components do not need to be disturbed due to their low permeability and magnetic susceptibility [53]. As a result, the current–voltage (I-V) characteristics of LEDs indicate no interruption based on comparison of two curves from the device with and without the magnetic field (Fig. 4h). In addition, p-n diodes and metal–oxide–semiconductor field-effect transistors (MOSFET) (Fig. S13), and gyroscopes (Figs. 4i and S14) also exhibit stable performances in the measured characteristics under applied B-field of 200 mT, allowing for in situ sensing on the integrated robots. Evaluation of the electrical stability of a system-on-chip under a typical range of magnetic strengths (~ 200 mT) also revealed negligible performance changes of less than 3%, which was also minimized (< 4%) even at high intensity (~ 400 mT) (Fig. S15).

### Multi-Modal Locomotion and Remote Operation of the Integrated Magnetic Soft Robot

**Fig. 5:**
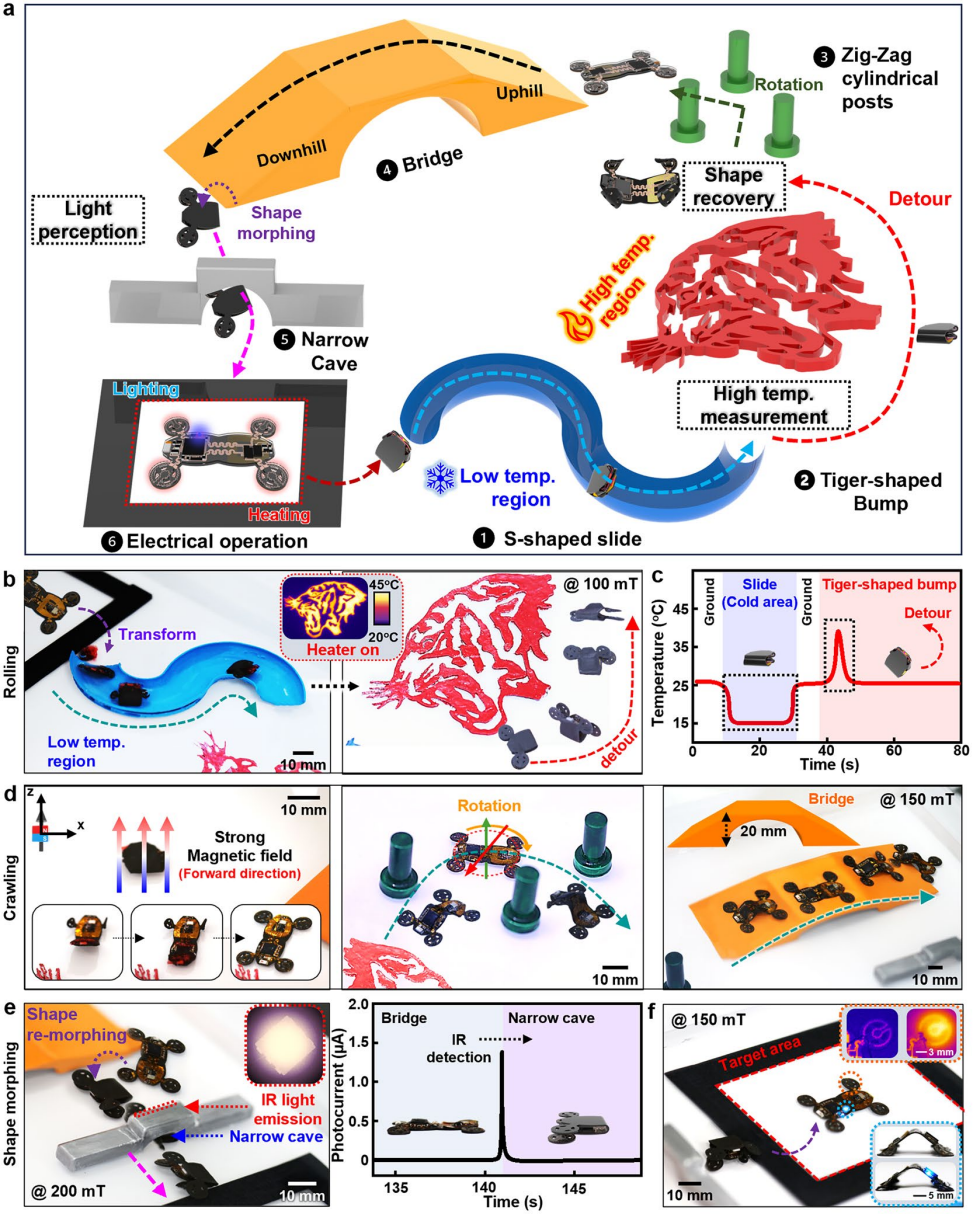
Demonstration of multi-modal locomotion and electrical functions of the integrated magnetic soft robot. **a** Schematic illustration of a variety of artificial obstacles to test locomotion/metamorphosis and electrical operation of the integrated magnetic soft robots. **b** Perception of temperature to morph the robot into a folded state and detour obstacles. Forward rolls of the folded robot to pass through an S-shaped slide (width, 30 mm) (left) and detour a heated, uneven tiger pattern (temperature: 45 °C) (right). **c** Real-time recording of temperature changes during both the movements. **d** Shape restoration of the integrated magnetic soft robot to the pre-programmed form (left), followed by passing through cylindrical posts (middle) and crawling across a bridge with uphill and downhill slopes (right). **e** Transformation back into the folded state and passing through a narrow cave (20 mm × 15 mm) when infrared (IR) was detected (left), and perceived profile of photocurrents by the integrated magnetic soft robot (right). **f** Arrival at the target area and remote optical and thermal operations as examples

Demonstration of electronics-integrated soft robot that can manage various, complex structures and environments in real time, suggests the possibility of practical uses. Figure 5a illustrates an artificial track to highlight on-demand, versatile transformations of a soft robotic system designed to sense atypical temperatures and illumination. The external magnetic field distribution on the experimental conditions appears in Fig. S16. The robot in Fig. 5b was deformed to a folded state in response to low-temperature area and rolled over a narrow S-shape slide (left). When encountering a hot section, the soft robot bypassed the heated tiger-shaped obstacle (right), and various parameters in surroundings can be detected in a continuous, real-time mode (Fig. 5c and Movie S3). The robotic system was then returned to the original shape (left, Fig. 5d), to escape from cylindrical posts installed in a zigzag manner (middle) and to cross a bridge with uphill and downhill slopes (right). When sensing IR emission (~ 850 nm) from a narrow gate, the integrated magnetic soft robot underwent a metamorphosis into the folded state to pass through the confined space (left, Fig. 5e), and the corresponding emission profile was recorded as shown in the right frame of Fig. 5e. In Fig. 5f, the system still provided stable electrical/optical operations after diverse, repetitive transformations, and locomotion (demonstration of the integrated magnetic soft robot on the racetrack in wet conditions is shown in Fig. S17 and Movie S4).

## Conclusions

The concepts, materials, design, and demonstrations reported here propose a soft, untethered electronic robot that can control diverse modes of locomotion/movement/actuation and perform active/passive electrical, thermal, and optoelectrical functions. The soybean wax was engineered with magnetic nanoparticles to lower the phase transition temperature, which facilitated magnetization or re-programming of the magnetic composites in a facile manner without high thermal energy and damages to the polymeric matrix. Mechanical and magnetic characterizations of the composites revealed that the modulus and magnetic field strength can be manipulated with concentrations of the magnetic filler and physical dimensions of the elastomeric matrix. Integrated wireless circuit and device components in the sophisticated design layout that does not interfere with locomotion or other motions, provided real-time information wirelessly via measuring or actuating various parameters. Such soft electronic robot was able to navigate diverse types of obstacles and conduct wireless electrical/optical operations, which paves the way to enhance or expand the functionality of established soft robots and to create applications for environmental monitors or medical implants using non-toxic materials-based elements with miniaturization of the whole system.

## Supplementary Information

Below is the link to the electronic supplementary material.

Supplementary file1 (MP4 12960 KB)

Supplementary file2 (MP4 13560 KB)

Supplementary file3 (MP4 8256 KB)

Supplementary file4 (MP4 21383 KB)

Supplementary file5 (DOCX 7305 KB)
