# Supplementary material for: Wireless, Multifunctional System-Integrated Programmable Soft Robot
Source: Nanomicro Lett. 2025 Feb 17;17:152. doi: 10.1007/s40820-024-01601-3 (PMC11832865; doi:10.1007/s40820-024-01601-3)
Supplement: Supplementary file 5 — Supplementary file5 (DOCX 7305 KB) [file 40820_2024_1601_MOESM5_ESM.docx]

Supporting Information for

**Wireless, Multifunctional System-integrated Programmable Soft Robot**

Sungkeun Han^1,‡^, Jeong-Woong Shin^1,2,‡^, Joong Hoon Lee^1,3,‡^, Bowen Li^4,‡^, Gwan-Jin Ko^1^, Tae-Min Jang^1^, Ankan Dutta^4,5^, Won Bae Han^1^, Seung Min Yang^1,6^, Dong-Je Kim^1^, Heeseok Kang^1^, Jun Hyeon Lim^1^, Chan-Hwi Eom^1^, So Jeong Choi^1^, Huanyu Cheng^4,7,8,*^, and Suk-Won Hwang^1,9,10,*^

^1^KU-KIST Graduate School of Converging Science and Technology, Korea University, 145 Anam-ro, Seongbuk-gu, Seoul, 02841, Republic of Korea

^2^Semiconductor R&D Center, Samsung Electronics Co., Ltd., Hwaseong-si, Gyeonggi-do, 18448, Republic of Korea

^3^SK Hynix, Icheon, 17336, Republic of Korea

^4^Department of Engineering Science and Mechanics, The Pennsylvania State University, University Park, PA 16802, USA

^5^Center for Neural Engineering, The Pennsylvania State University, State College, University Park, PA, 16802, USA

^6^Hanwha Systems Co., Ltd., 188, Pangyoyeok-ro, Bundang-gu, Seongnam-si, Gyeonggi-do, 13524, Republic of Korea

^7^Department of Materials Science and Engineering, The Pennsylvania State University, State College, University Park, PA 16802, USA

^8^Materials Research Institute, The Pennsylvania State University, State College, University Park, PA 16802, USA

^9^Center for Biomaterials, Biomedical Research Institute, Korea Institute of Science and Technology (KIST), Seoul 02792, Republic of Korea

^10^Department of Integrative Energy Engineering, Korea University, Seoul 02841, Republic of Korea

^‡^Sungkeun Han, Jeong-Woong Shin, Joong Hoon Lee, and Bowen Li contributed equally to this work.

*Corresponding authors. E-mail: [dupong76@korea.ac.kr](mailto:dupong76@korea.ac.kr) (Suk-Won Hwang); [Huanyu.Cheng@psu.edu](mailto:Huanyu.Cheng@psu.edu) (Huanyu Cheng)

**S1 Supplementary Methods**

**Method S1 Programming sequence of soft WcMPs composites**

Soft WcMPs composites are programmed into various shapes through three steps: Initially, the WcMPs composites are heated above the transition temperature (approximately 45 ^o^C), in order to liquefy the wax shell. Following this, the external magnetic fields are employed to align the magnetic particles within the elastomer matrix. Finally, the WcMPs composites are cooled to solidify the wax shell and pin the magnetic moments.

**Method S2 Theoretical calculation of deflection at free ends of the magnetic soft composites**

When an applied magnetic field deviates from alignment with the magnetization direction of the composite's magnetic particles, it induces a magnetic torque (τ) to realign them. The generated magnetic torque, causing elastic deformation, is described by

$$\begin{aligned} \tau=M\times B\#\left( S1 \right) \end{aligned}$$

where B, M denotes the applied magnetic flux density and the magnetization value of the composite. Using standard beam equation and boundary condition, deflection (Δ) caused by external magnetic field can be described as

$$\begin{aligned} \Delta= \left( \frac{MB}{G} \right)\left( \frac{L}{A} \right)^{2}\#\left( S2 \right) \end{aligned}$$

where G, L, and A indicates shear modulus in the moony model, length of the composite beam, and area of cross-sectional beam.

**S2 Supplementary Figures**


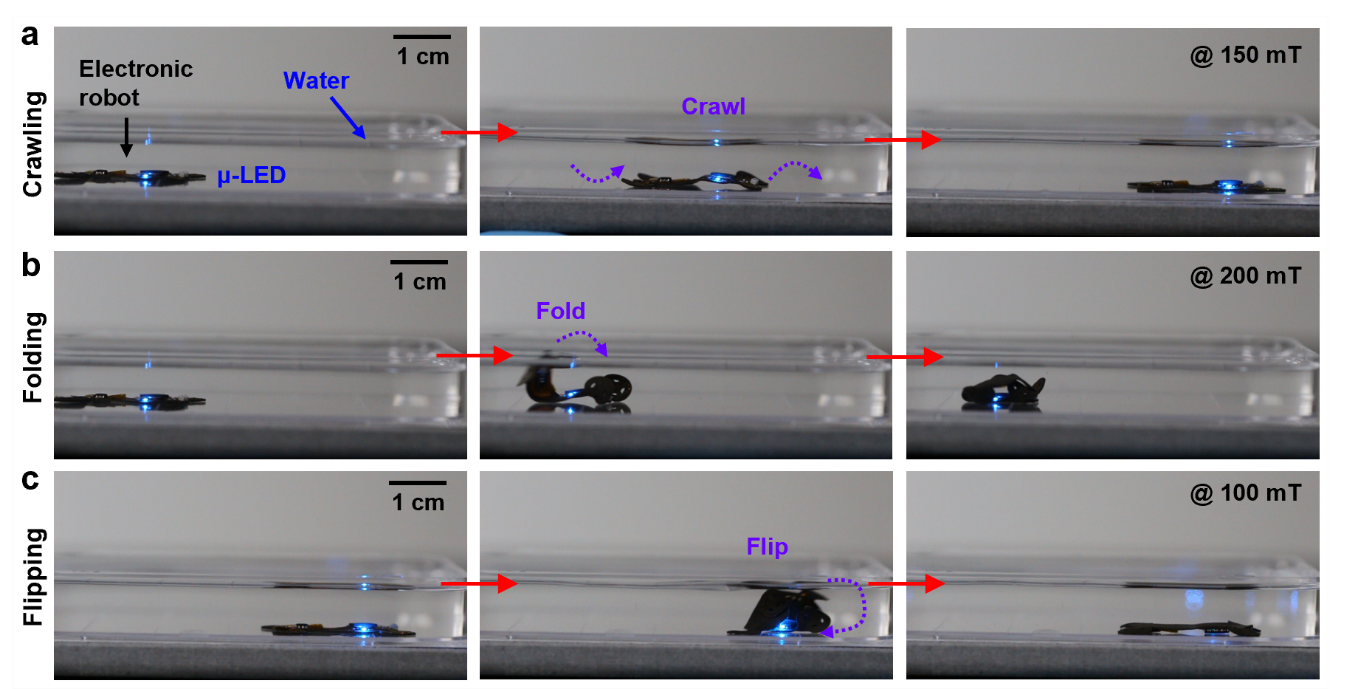


**Fig. S1 Various actuation performances in the water terrain.** **a-c** Sequential images illustrating the locomotion of the integrated soft robot in an aquatic environment, including crawling (**a**), folding (**b**), and flipping (**c**)


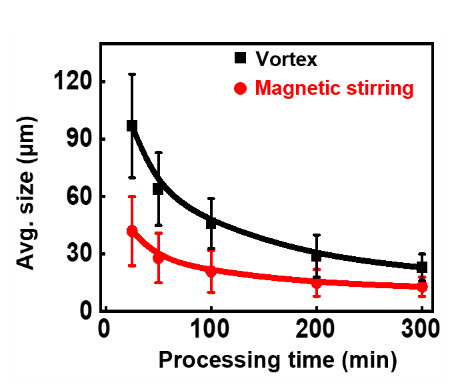


**Fig. S2 Distribution of averaged size of WcMPs over mixing time with vortex and magnetic stirring**


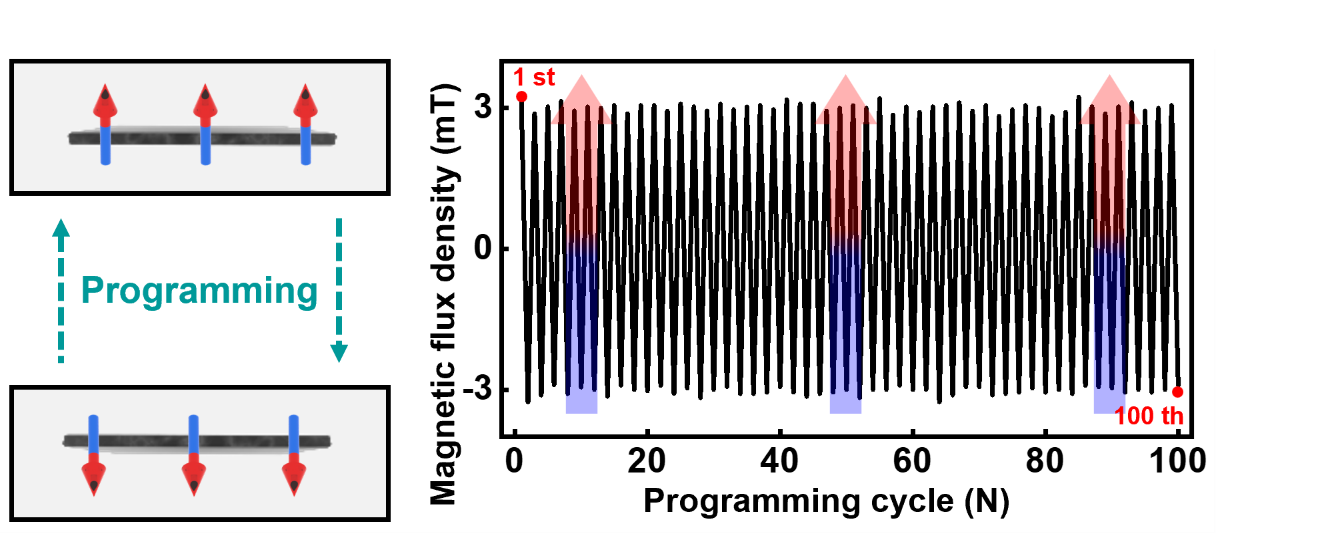


**Fig. S3 Magnetic flux density changes of the magnetic soft robot during repeated programming process**


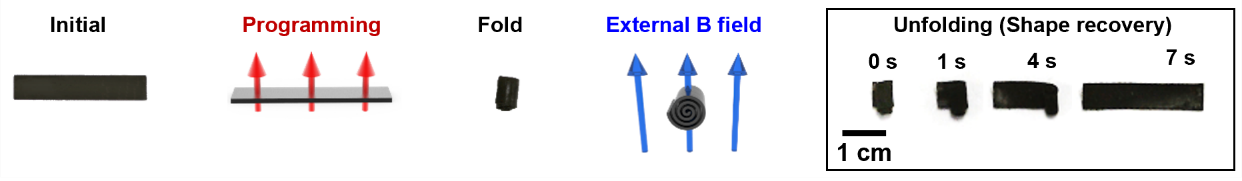


**Fig. S4 Shape morphing performance of complex deformed beam-like magnetic soft matrix under an external magnetic field (150 mT)**


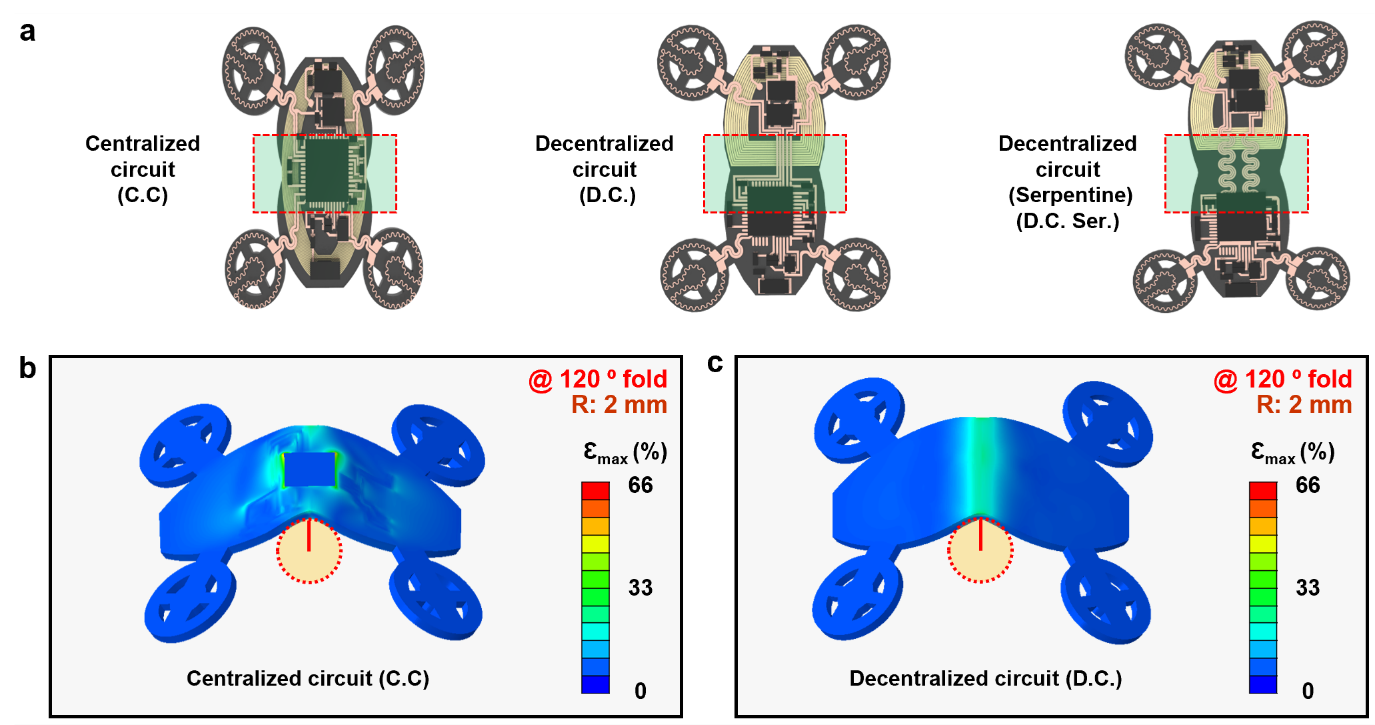


**Fig. S5 Integrated electronic system layouts.** **a** Schematic illustration of the integrated electronic system layouts: centralized circuit (C.C.) (left), decentralized circuit (D.C.) (middle), decentralized circuit with serpentine interconnect (D.C.Ser.) (right). **b, c** Strain distribution at 120-degree folding with the radius of curvature at 2 mm for the robot system with C.C. (**b**) and D.C. (**c**)


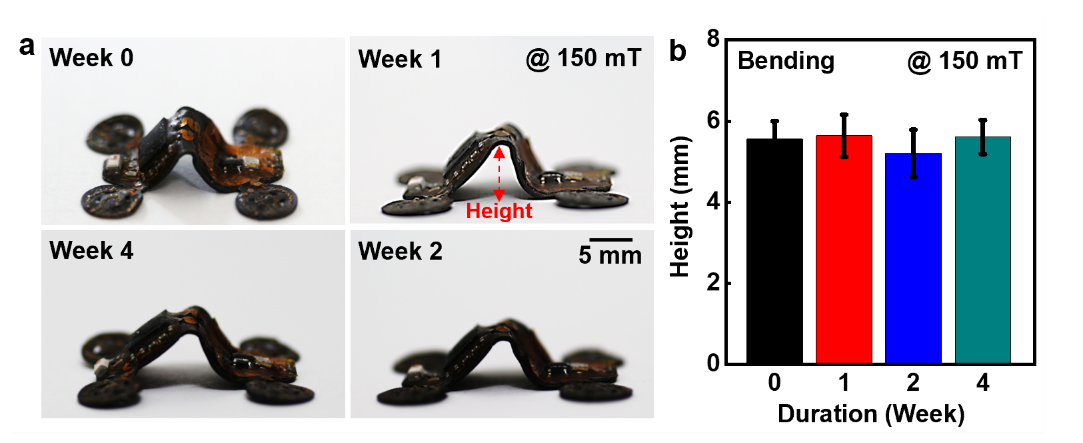


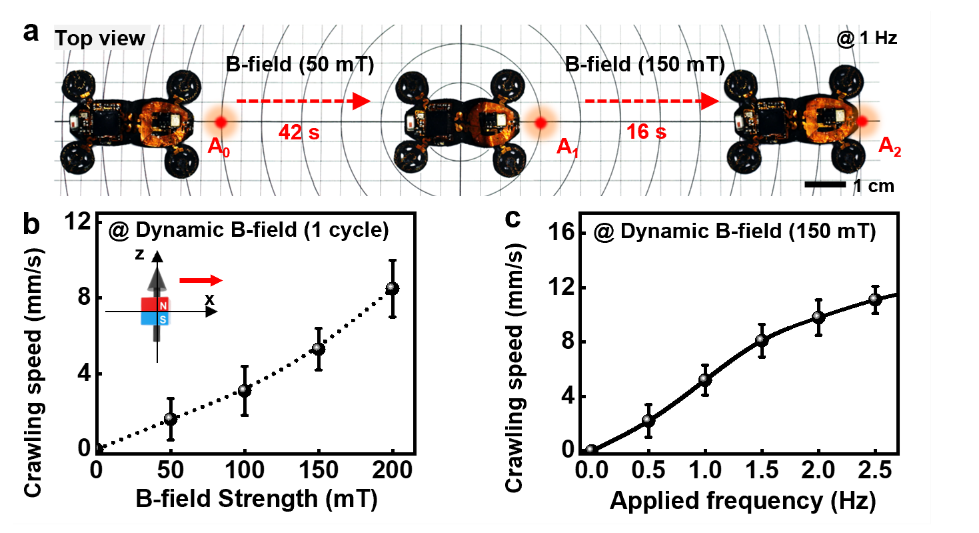
**Fig. S6** **Evaluation of long-term actuation stability of the magnetic soft robot with D.C. Ser system layout.** **a, b** Demonstration of height at bending area of magnetic robot during crawling locomotion (**a**), and evaluation of the height difference (**b**) over 4 weeks

**Fig. S7 Crawling performance of the integrated magnetic soft robot.** **a** Time-lapse measurement of displacement demonstrated that travel time of integrated magnetic soft robot is inversely proportional to the magnetic field (A_0_ to A_1_, 42 sec at 50 mT; A_1_ to A_2_, 16 sec at 150 mT). **b** Experimentally measured the crawling speed on a one-time actuation with different magnetic field strengths. **c** Experimentally evaluated crawling speed over various frequencies of magnetic field (150 mT)


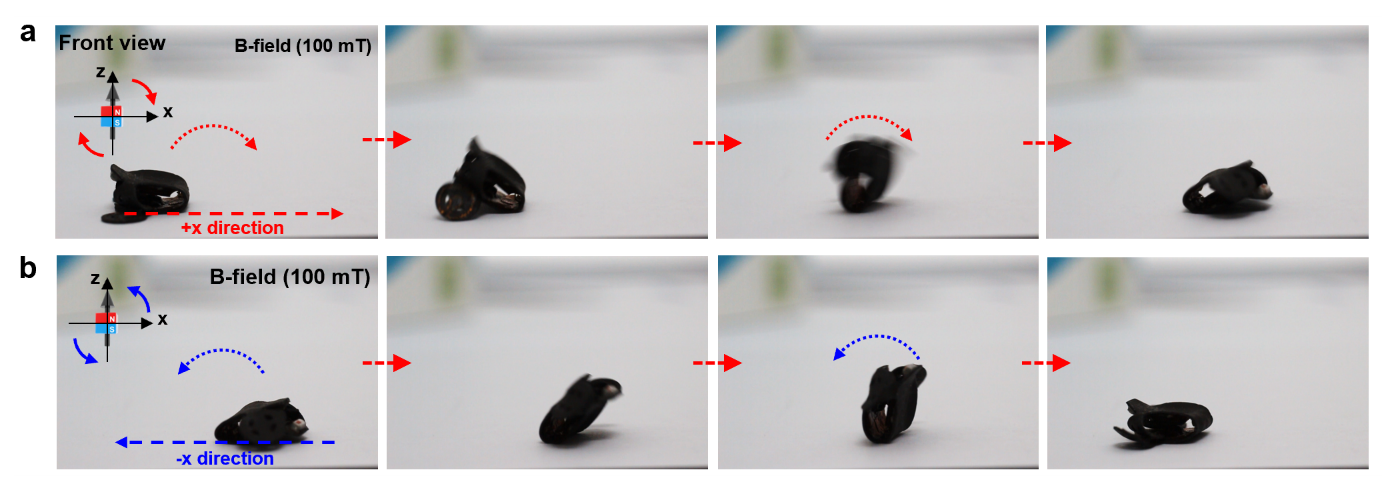
**Fig. S8 Rolling locomotion of the integrated magnetic soft robot.** A series of images demonstrating the side-to-side rolling behavior of an integrated magnetic soft robot as a function of the rotational direction of the external magnetic field. **a** Forward. **b** Backward


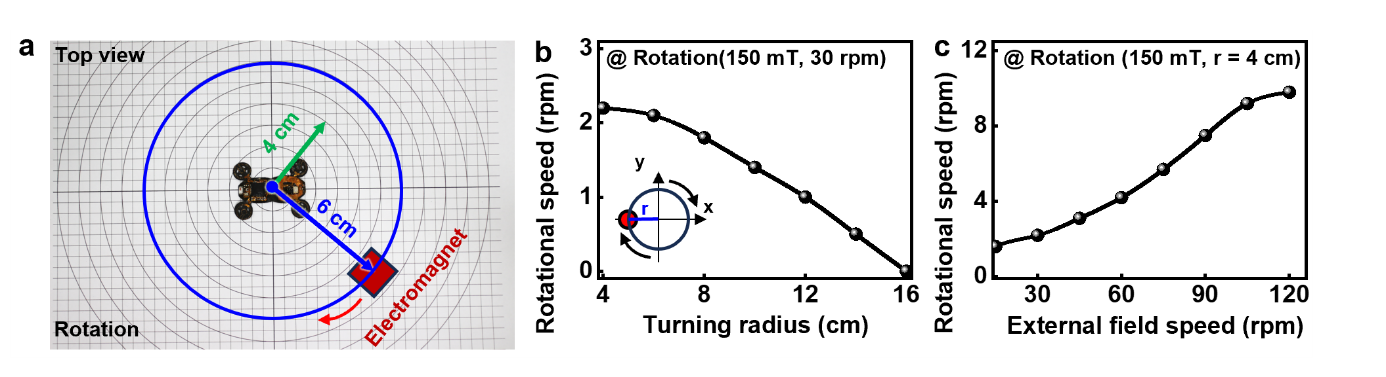


**Fig. S9 Rotational movement of the integrated magnetic soft robot during clockwise revolution of periodic magnetic field.** **a** Optical image presenting the procedure of the rotational locomotion. **b, c** Measured rotational speed of the integrated magnetic soft robot as each function of the turning radius of magnetic source (**b**) (150 mT, 30 rpm) and turning speed of magnetic source (**c**) (150 mT, r: 4 cm)


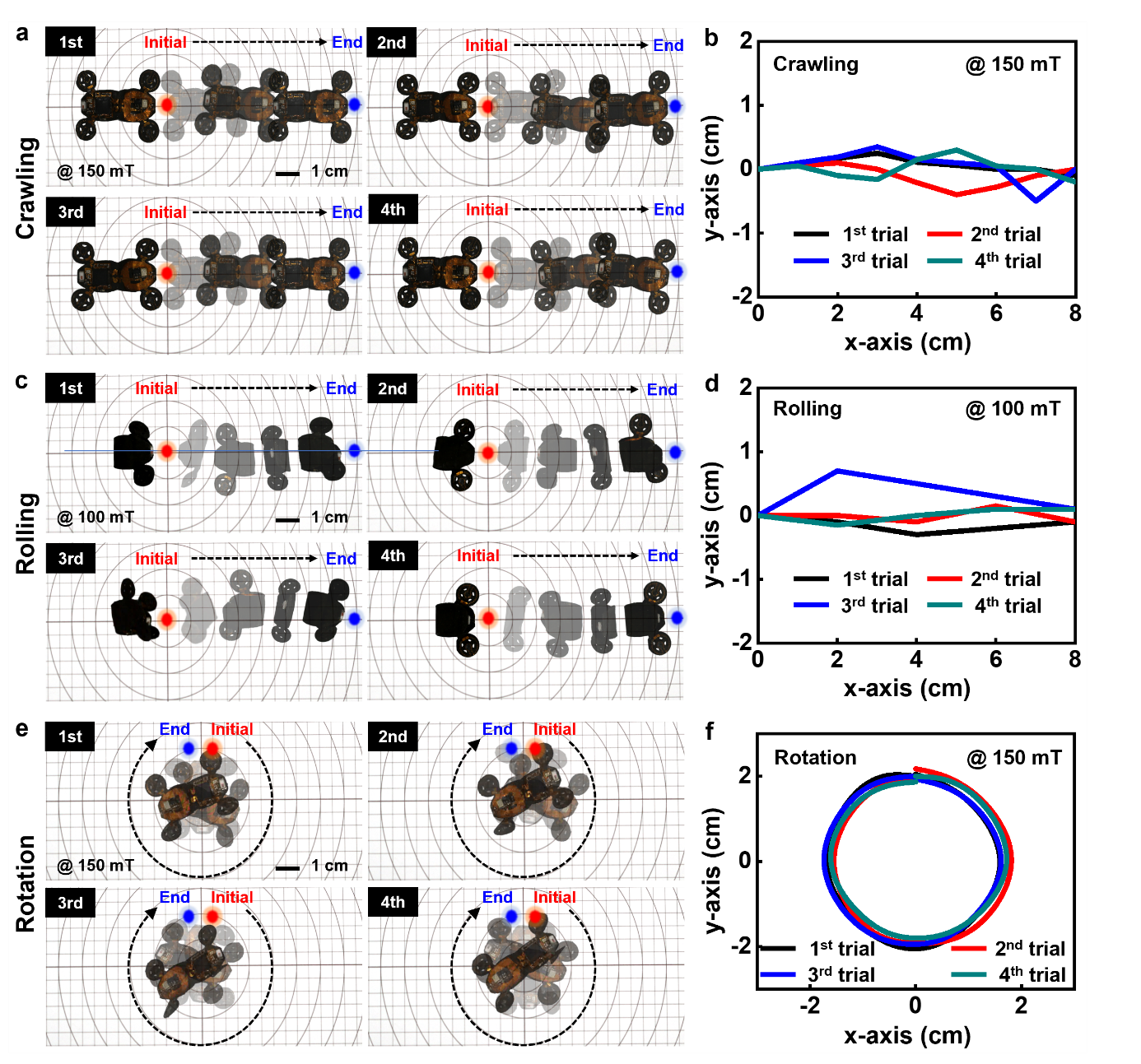


**Fig. S10 Validation of the trajectory tracking of the integrated magnetic soft robot.** **a** A series of images of the crawling and **b** a plot of changes in the path. **c** A Sequence of images of the rolling and **d** estimated travel route. **e** A set of images of the rotation and **f** analysed turning track


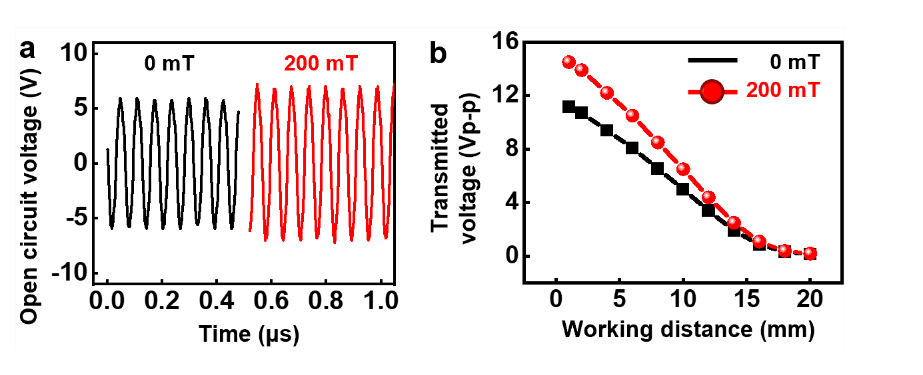


**Fig. S11 Effect of magnetic field on the performance of Cu inductive coil.** **a** Variation in open-circuit voltage of Cu coil under external magnetic field (200 mT). **b** Changes in peak-to-peak voltage at different working distances for Cu coil under external magnetic field (200 mT)


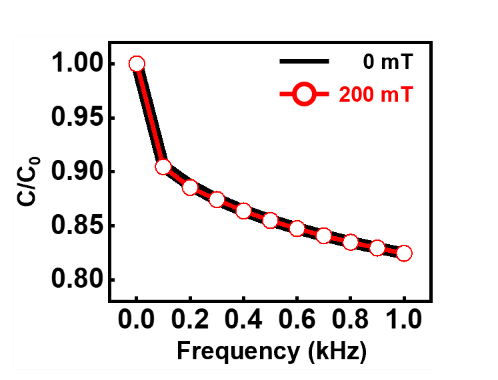


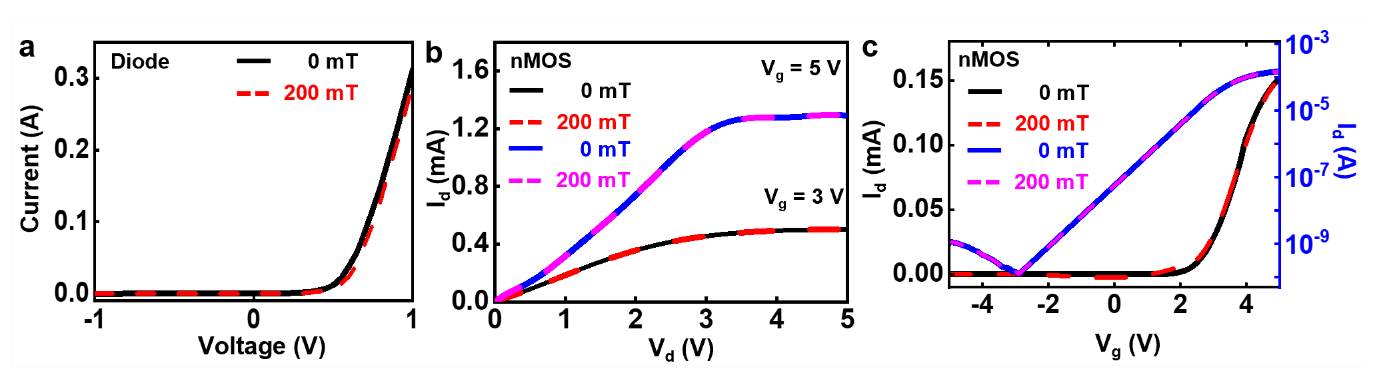
**Fig. S12 Measured capacitance changes in capacitors under external magnetic field**

**Fig. S13 Comparison of current-voltage characteristics under magnetic field.** **a** I-V curve of p-n diode. **b** Output curve of n-MOSFET. **c** Transfer curve of n-MOSFET


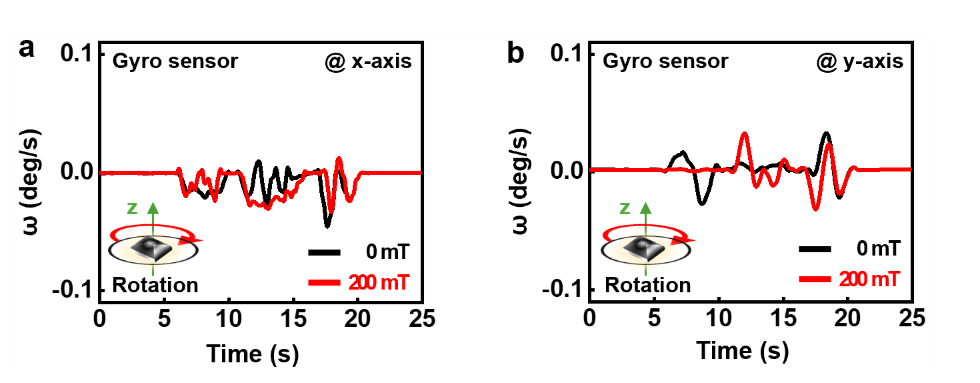


**Fig. S14 Evaluation of the effect of a magnetic field on the performance of gyro sensors rotating along the z-axis**


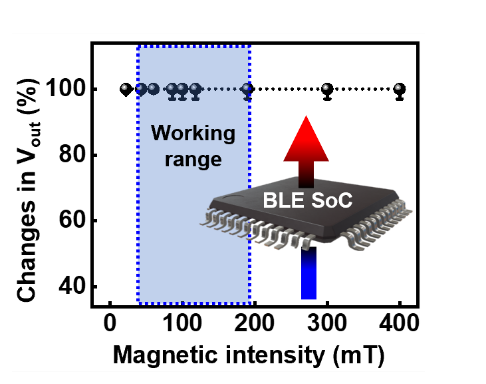


**Fig. S15 Assessment of the electrical stability of system-on-chip under a variety of levels of external magnetic fields**


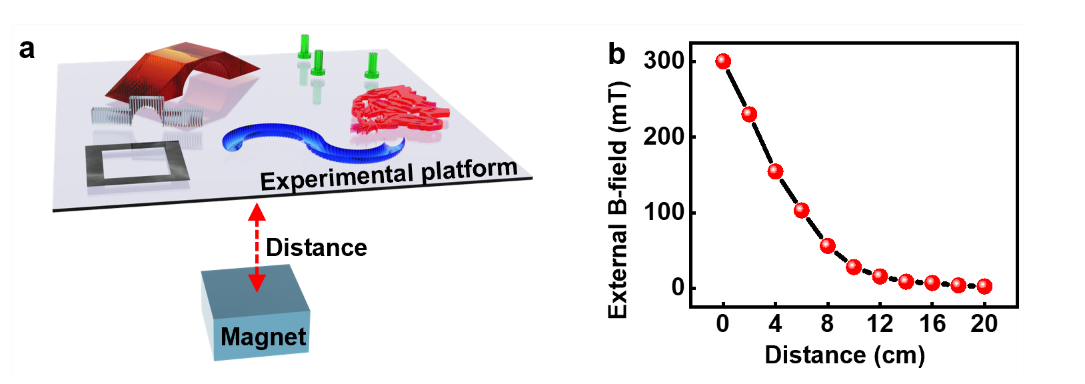


**Fig. S16 The external magnetic field distribution on the experimental platform.** **a** Schematic illustration of the experimental platform with magnetic source. **b** External magnetic field distribution along with distance between platform and magnetic source

**
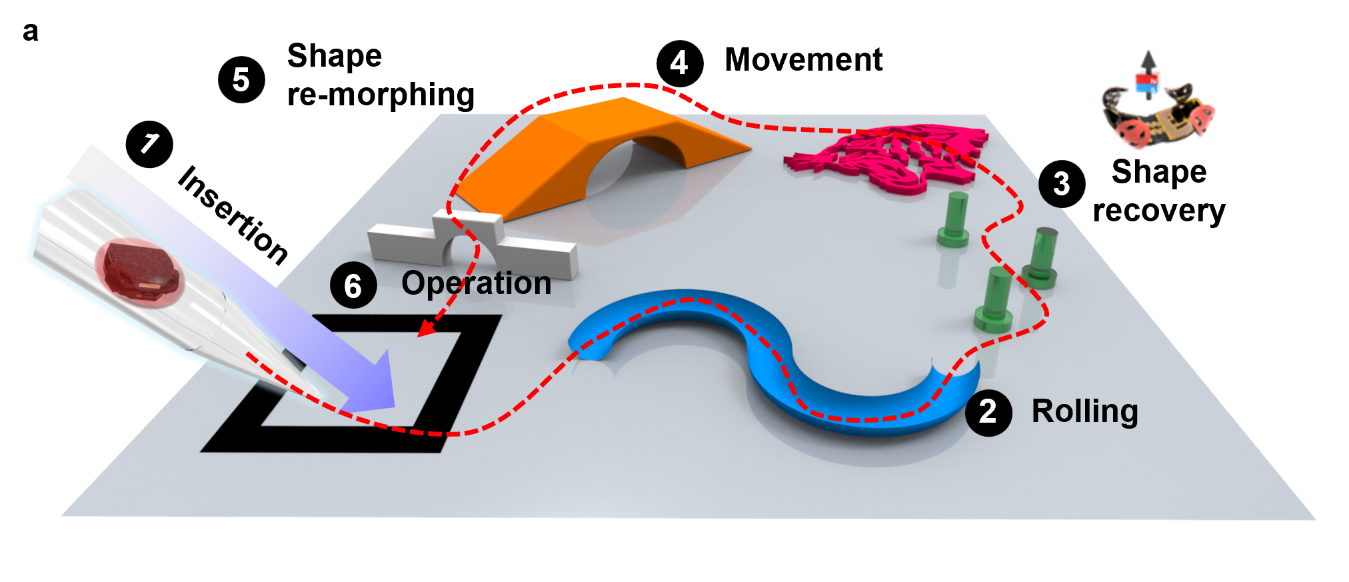
**

**Fig. S17 Schematic illustration showing different types of artificial barriers used to evaluate the movement, shape morphing abilities, and electrical performance of soft electronic robots (wet condition)**

**S3 Supplementary Movies**

**Movie S1 Shape morphing performance**

**Movie S2 Relative actuation performance according to electronic circuit layouts**

**Movie S3 *In situ* temperature monitoring**

**Movie S4 Demonstration of multi-modal locomotion and functions of integrated magnetic soft robot**
